# Variations in positive well-being as a function of the interaction between paranormal belief and schizotypy

**DOI:** 10.3389/fpsyg.2024.1396485

**Published:** 2024-07-26

**Authors:** Neil Dagnall, Kenneth Graham Drinkwater, Andrew Denovan, Alex Escolá Gascón

**Affiliations:** ^1^Department of Psychology, Manchester Metropolitan University, Manchester, United Kingdom; ^2^School of Psychology, Liverpool John Moores University, Liverpool, United Kingdom; ^3^Department of Quantitative Methods and Statistics, Comillas Pontifical University, Madrid, Spain

**Keywords:** paranormal belief, schizotypy, well-being, paranormal experience, belief in conspiracies, latent profile analysis

## Abstract

This study examined variations in positive well-being as a function of paranormal belief and schizotypy. A sample of 2,362 United Kingdom-based respondents completed self-report measures assessing paranormal belief, schizotypy, positive well-being (meaning in life, satisfaction with life, and self-esteem), paranormal experience, and belief in conspiracies. The paranormal belief was most strongly related to the cognitive–perceptual factor of schizotypy. Both paranormal belief and the cognitive–perceptual factor were associated with reporting paranormal experiences and endorsement of conspiracist beliefs. Despite commonality, paranormal belief and schizotypy were differentially related to well-being. Paranormal belief correlated positively with meaning in life (presence and search) and satisfaction with life. Schizotypy correlated negatively with presence, satisfaction with life, and self-esteem and positively with search. Latent profile analysis identified four subgroups: Profile 1, low belief and schizotypy (49% of the sample); Profile 2, low belief and cognitive–perceptual, moderate interpersonal and disorganised (13.6%); Profile 3, high belief, moderate cognitive–perceptual and interpersonal, low disorganised (24.3%); and Profile 4, high belief and schizotypy (13.1%). Multivariate analysis of variance (MANOVA) found that low belief with mixed schizotypy was associated with lower presence, and low belief and schizotypy (vs. high) were related to higher presence. Paranormal belief and schizotypy were associated with greater search, higher scores on paranormal experiential factors, and endorsement of generic conspiracist beliefs. Finally, lower belief and schizotypy were concomitant with higher satisfaction with life and self-esteem. Overall, paranormal belief was related to positive well-being, whereas schizotypy was associated with lower positive wellbeing.

## Introduction

Surveys consistently report that paranormal belief persists within modern Western societies (Drinkwater et al., 2021a). Illustratively, a 2005 Gallup survey found that 73% of Americans endorsed belief in at least one paranormal phenomenon (Moore, 2005). A sizeable proportion of the population also claimed to have experienced supernatural phenomena (see Dagnall et al., 2016). As researchers have operationalised paranormality in diverse ways, this paper adopted the delineation of Irwin (2009). This states that ‘a paranormal belief is defined on a working basis as a proposition that has not been empirically attested to the satisfaction of the scientific establishment but is generated within the non-scientific community and extensively endorsed by people who might normally be expected by their society to be capable of rational thought and reality testing’ (Irwin, 2009, p. 16, 17).

Despite the enduring popularity of supernatural credence, researchers typically regard beliefs as maladaptive and/or indicative of soft psychopathology symptoms (Dagnall et al., 2022a). This pejorative view prevails even though the evidence is inconsistent (i.e., dated and focused on restricted facets of belief such as superstition) and derives from correlations between overall belief and variables allied to poor psychological adjustment (e.g., depressive attributional style and increased negative emotional states; Dudley and Whisnand, 2000).

Assuming paranormal powers/forces do not exist, the everyday nature of belief in general populations suggests that it is best conceptualised as an individual difference (i.e., a continuous quantifiable variation) rather than a distinct point of classification. Accordingly, personal endorsement arises from limited consideration of empirical evidence. This is especially true, as corroborating substantiation is limited (e.g., Bem, 2011) and criticised by the scientific community (Wagenmakers et al., 2011).

Acknowledging this, cognitive theorists view paranormal beliefs as a manifestation of preferential thinking style and/or truncated reasoning (Williams et al., 2022). For instance, dual-processing explanations ascribe validation to an inclination for subjective/affect-based (vs. objective/logic) data. The advantage of the cognitive approach is that it regards paranormal belief as domain-specific. Believers can process/reason at a high evaluative level but employ flawed reasoning in the context of the paranormal. Hence, errors arise because judgements derive from personal (rather than objective) appraisal of evidence (Dagnall et al., 2010a, 2015).

Irwin (2003) referred to this flawed decision-making process as reality testing failure. Within this conceptualisation, belief and mentation are reciprocally reinforcing and provide a skewed interpretive lens (Irwin et al., 2013; Lange et al., 2019). This is also commensurate with the notion that supernatural credence is a form of subclinical psychosis (e.g., Van Os, 2003; Unterrassner et al., 2017), whereby characteristics found in psychosis (magical thinking, ideas of reference, unusual experiences, etc.) present in a milder form within general populations (Unterrassner et al., 2017). Correspondingly, paranormal beliefs/experiences represent non-clinical delusions and hallucinations (Irwin et al., 2012a,b).

Recent research designates that supernatural credence is not necessarily related to poor psychological adjustment/well-being. Whilst there is correlation-based evidence to support the assertation that paranormal belief is associated with reduced well-being, this ignores the heterogeneous nature of believers and the fact that personal credence is qualified by the presence of other psychological factors (e.g., schizotypy; Denovan et al., 2018). Interactions between belief and these factors differentially influence relationships with well-being outcomes. Testing this, Drinkwater et al. (2024) assessed whether subgroup membership, determined by paranormal belief and psychopathology (schizotypy and manic-depressive experience), predicted well-being (i.e., stress, somatic complaints, life satisfaction, and meaning in life) over time. The analysis found that the highest psychopathology scoring profile (vs. lower) predicted higher negative and lower positive well-being. Transliminality and fearful attitude (positively) and sceptical attitude (negatively) mediated this outcome. Thus, in the absence of psychopathology, paranormal belief had no influence on well-being.

In a related study, Dagnall et al. (2022c) reported that higher scores on transliminality and psychopathology-related variables (i.e., the unusual experiences and cognitive disorganisation subscales of schizotypy and manic-depressive experiences) were associated with stronger paranormal belief and somatic complaints relationship. Furthermore, as levels of transliminality and unusual experiences increased, the strength of the relationship between paranormal belief and perceived stress also increased. These outcomes demonstrated that cognitive–perceptual personality factors qualified the effect of supernatural credence.

In the case of transliminality, higher scores reflect reduced ability to suppress irrelevant information (Thalbourne, 2009). Regarding unusual experiences, positive symptoms (e.g., magical thinking, perceptual aberrations, and hallucinations) are expressed as broadening cognitions (e.g., hallucinations and paranoia). Combined, transliminality and unusual experiences increase perceptual sensitivity to stimuli and decrease the ability to discriminate between internally and externally generated data (Dagnall et al., 2008). This explains why the presence of these constructs, alongside paranormal belief, amplifies awareness of somatic complaints and stress.

A study by Dagnall et al. (2022a), using network analysis, further demonstrated the influential effects of transliminality, unusual experiences (positive schizotypy), and depressive experiences on the paranormal belief well-being relationship. Transliminality acted as a connecting variable between belief, positive schizotypy, and psychopathology. Additionally, depressive experiences bridged the transliminality and well-being relationship.

The finding that levels of cognitive–perceptual factors qualify paranormal beliefs concurs with studies using latent profile analysis (LPA). Dagnall et al. (2022b) identified subgroups using paranormal belief and psychopathology (schizotypy, depression, manic experience, and depressive experience) and examined differences in well-being scores (i.e., perceived stress, somatic complaints, and life satisfaction). Subgroups with higher psychopathology reported lower well-being. However, higher paranormal belief was not necessarily associated with lower psychological adjustment and reduced well-being. These results indicated that belief in the context of well-being was not maladaptive.

Noting the benign influence of paranormal belief on negative well-being, the present study explored whether credence had adaptive benefits. This was important since few studies have examined the positive effects of belief. Moreover, studies reporting benefits have typically focussed on experience (Kennedy and Kanthamani, 1995), employed small samples (e.g., Parra and Corbetta, 2014), and/or used qualitative approaches (Betsch et al., 2021). Whilst these reports enhanced well-being (e.g., increased happiness, confidence, optimism, and meaning in life), researchers have not established the degree to which outcomes extrapolate to broader, general populations.

Noting these factors, the present study, alongside direct relationships, used LPA to examine belief-related differences in perceived well-being. Based on previous research, LPA amalgamated paranormal belief with schizotypy. Corresponding with studies using general populations, the authors conceptualised schizotypy as a personality trait, assessed on a continuum, ranging from psychological health to schizophrenia (psychosis; Barrantes-Vidal et al., 2015). The advantage of this delineation is that it recognises that schizotypy is both a source of healthy variation and an indicator of susceptibility to psychosis. Hence, schizotypy comprises facets that approximate features of schizophrenia or schizotypal personality disorder (Kwapil and Barrantes-Vidal, 2015).

To facilitate conceptual understanding of profiles, the study adopted the three-factor model proposed by Raine et al. (1994). Within this, there is a clear correspondence between factors and clinical symptoms. Cognitive–perceptual factors align with positive symptoms (i.e., delusions and hallucinations), specifically odd beliefs and magical thinking, unusual perceptual experiences, ideas of reference, and paranoid ideation. Interpersonal encompasses features affiliated with negative symptoms (i.e., social anxiety, constricted affect, paranoia, and no close friends). Finally, disorganised (i.e., odd speech and odd behaviour) reflects thought disorder and bizarre behaviour (Arndt et al., 1991).

The three-factor model was used in preference to the four-factor model (Mason, 2015) because it provides a narrower definition of schizotypy, which more easily maps on to classically defined symptomology (i.e., positive, cognitive disorganisation, and negative, see Kemp et al., 2021). This is advantageous in terms of parsimonious identification of profiles. To enable comparisons with prior investigations (Kennedy and Kanthamani, 1995; Parra and Corbetta, 2014; Betsch et al., 2021), meaning in life, satisfaction with life, and self-esteem were employed as well-being indices.

This study built on previous research in two notable ways. First, it established whether interactions between paranormal belief and schizotypy produced profiles similar to those described in previous LPA studies (e.g., Denovan et al., 2018). This was particularly important because alternative measures of schizotypy exist. As previous studies (e.g., Denovan et al., 2018; Drinkwater et al., 2024) have typically employed the Oxford–Liverpool Inventory of Feelings and Experiences (O-LIFE short; Mason et al., 2005), the present study used the Schizotypal Personality Questionnaire—Brief (SPQ-B; Raine and Benishay, 1995).

Second, the investigators included a broader range of positive well-being measures, a further development is a separate analysis of meaning in life dimensions (i.e., presence and search). This was important because presence correlates positively with affirming outcomes (e.g., life satisfaction and joy) and negatively with adverse indicators (e.g., depression and sadness; Steger et al., 2006), whereas search is associated with negative emotions (Cohen and Cairns, 2012) and reduced well-being (e.g., depression, sadness, and rumination; Dakin et al., 2021).

Consistent with previous findings, the authors predicted that positive well-being outcomes would correlate positively with paranormal belief and negatively with schizotypy. In line with these hypotheses, the authors anticipated that relationships between emergent profiles and well-being outcomes would vary as a function of levels of belief and schizotypy. To ensure that profiles possessed conceptual coherence, the study included an assessment of paranormal experience and belief in conspiracies. As these factors correlate positively with both supernatural credence and schizotypy, especially the cognitive–perceptual dimension, the authors anticipated that profiles with higher levels of these constructs would report more paranormal experiences and greater conspiratorial endorsement.

## Materials and methods

### Participants

A sample of 2,362 respondents participated in this study (*M*age = 46.73, *SD* = 12.97, and range = 18 to 82). There were 1,244 men (*M*age = 47.83, *SD* = 12.70, and range = 18 to 78), 1,103 women (*M*age = 45.61, *SD* = 13.12, and range = 18 to 82), 13 non-binary (*M*age = 37.61, *SD* = 14.08, and range = 18 to 59), and 2 did not disclose gender identity (*M*age = 39.0, *SD* = 29.69, and range = 18 to 60). The researchers recruited participants through Bilendi, which is an acknowledged supplier of quality data (Fladerer and Braun, 2020). The use of participation pools is equivalent to traditional recruitment measures (Kees et al., 2017). The advantages are the ability to sample a broader range of ages and control for gender bias. The researchers hosted the survey within Qualtrics (a web-based software) and participants accessed it via a web link. Bilendi disseminated the link to members of their participation pool in accordance with the researcher’s instructions. These stipulated a United Kingdom-based, gender-balanced sample with a minimum participant age of 18 years. Bilendi provides data from established respondent pools, where individuals have consented to participate in online research studies.

### Measures

The study employed established self-report measures. The investigators collated this to produce an online survey. Latent profiles comprised paranormal beliefs and schizotypy.

#### Revised Paranormal Belief Scale

The Revised Paranormal Belief Scale (RPBS, Tobacyk, 2004) contains 26 items that represent core facets of paranormal belief (i.e., traditional religious belief, psi belief, witchcraft, spiritualism, superstition, extraordinary life forms, and precognition; see Dagnall et al., 2010b). Items appear as statements (e.g., ‘It is possible to communicate with the dead’), and respondents answer using a 7-point Likert-type scale, ranging from 1 (strongly disagree) to 7 (strongly agree) (Tobacyk, 2004). Prior to analysis, consistent with Irwin (2009), responses were recoded (0–6). Hence, scores range from 0 to 156. Higher scores reflect greater paranormal belief.

#### The Schizotypal Personality Questionnaire—Brief

The Schizotypal Personality Questionnaire—Brief (SPQ-B, Raine and Benishay, 1995) is a 22-item instrument that assesses schizotypal personality features/disorders in non-clinical populations. The measure presents items as statements (e.g., ‘People sometimes find me aloof and distant’) and participants respond on a dichotomous scale (0 = No and 1 = Yes). The SPQ-B comprises three subscales: cognitive–perceptual (eight items), interpersonal (eight items), and disorganisation (six items). The SPQ-B produces subscale scores and an overall total of 0–22. Higher scores denote greater levels of schizotypy.

### Outcomes

#### Paranormal experience

Paranormal experience (PE) is a short 3-item measure that assesses personal involvement with the supernatural. Respondents indicate, using Yes/No responses, whether they have experienced paranormal phenomena, visited paranormal practitioners, or demonstrated paranormal abilities. The researchers developed the PE across a series of studies (e.g., Dagnall et al., 2016; Drinkwater et al., 2021b), and it has become an established research instrument (Drinkwater et al., 2021a, 2022). Item totals provide an overall indication of personal paranormal experience (0–3). Higher scores reflect greater engagement.

#### Generic Conspiracist Beliefs Scale—Short

The Generic Conspiracist Beliefs Scale—Short (GCB-5, Kay and Slovic, 2023) is a 5-item, abridged version of the Generic Conspiracist Beliefs Scale (Brotherton et al., 2013), which assesses the tendency to endorse general (i.e., non-event-based) conspiracy-related ideas and concepts (Kay and Slovic, 2023). Investigators have widely adopted the GCBS, and the instrument has attested psychometric properties (Brotherton et al., 2013; Drinkwater et al., 2020; Dinić et al., 2023). The GCB-5 is an expedient unidimensional measure designed for use in lengthy-scale batteries, to reduce respondent fatigue. The instrument comprises the highest loading items from the five GCBS dimensions: government malfeasance, extraterrestrial cover-up, malevolent global conspiracy, personal well-being, and control of information. Researchers have recently validated the GCB-5 (Dagnall et al., 2023; Kay and Slovic, 2023). Items appear as statements (e.g., ‘A small, secret group of people is responsible for making all major world decisions, such as going to war’) and participants respond by completing a 5-point Likert-type scale (1 = definitely not true to 5 = definitely true). Item summation produces an overall total (5–25), with higher scores being indicative of greater levels of generic conspiratorial ideation.

#### Meaning in Life Scale

The Meaning in Life Scale (MLS, Steger et al., 2006) is a 10-item instrument that assesses the presence of (5 items) and searches for (5 items) purpose in life. Presence denotes the extent to which respondents feel their life has meaning (e.g., ‘My life has a clear sense of purpose’). Search measures the degree to which individuals attempt to find or deepen life meaning (e.g., ‘I am always searching for something that makes my life feel significant’). Items appear as statements and participants record their responses on a 7-point Likert-type scale, ranging from 1 (‘absolutely untrue’) to 7 (‘absolutely true’). Higher scores reflect greater presence (5–35) and search (5–35).

#### Satisfaction with Life Scale

The Satisfaction with Life Scale (SWLS, Diener et al., 1985) is a 5-item measure of the cognitive component of subjective well-being that provides an integrated judgement of life satisfaction. The SWLS presents items as statements (e.g., ‘In most ways my life is close to my ideal’), and participants record responses on a 7-point Likert-type scale, ranging from 1 (‘Strongly Disagree’) to 7 (‘Strongly Agree’). Higher scores reflect greater satisfaction with life (5–35).

#### Rosenberg Self-Esteem Scale

The Rosenberg Self-Esteem Scale (RSE, Rosenberg, 1965) is a 10-item instrument that assesses global self-worth/acceptance. Participants respond to each item, which appears as statements (e.g., ‘I take a positive attitude towards myself’) via completion of a 4-point Likert-type scale (1 = Strongly Disagree to 4 = Strongly Agree). Scores range from 10 to 40, with higher values specifying greater self-esteem.

The measurement instruments selected were theoretically and psychometrically satisfactory (RPBS, Drinkwater et al., 2017; SPQ-B, Raine and Benishay, 1995; PEFs, Drinkwater et al., 2022; GCBS-5, Dagnall et al., 2023; Kay and Slovic, 2023; MLS, Steger et al., 2006; TSWLS, Guitard et al., 2022; RSE, Monteiro et al., 2022).

Within the present study, scales demonstrated internal reliability: RPBS, α = 0.96; SPQ-B (cognitive–perceptual, α = 0.77; interpersonal, α = 0.80; and disorganised, α = 0.76); GCB-5, α = 0.85; MLS (presence, α = 0.89; search, α = 0.92); SWLS (α = 0.93; and RSE, α = 0.90).

### Procedure

Following the receipt of the hyperlink, participants accessed the study information. Participants only continued if they provided informed consent. Those advancing completed a demographic section (age, preferred gender, and occupation) and then progressed to the scales. Instructions directed participants to read all items carefully and work at their own pace. To limit potential order and carryover effects, Qualtrics’ inbuilt randomiser function rotated scale order across participants. On completion of the survey, respondents accessed the study debrief.

As study data were cross-sectional, collected at one point in time, the researchers utilised procedural counters to reduce the likelihood of common-method variance. Particularly, instructions highlighted scale/construct differences. This created psychological distance between survey sections and emphasised construct uniqueness (Spector, 2019). To reduce the possibility of evaluation apprehension and social desirability, survey instructions also stated that there were no correct answers and that responses should reflect individual preferences/thoughts (Krishnaveni and Deepa, 2013).

### Ethics statement

The Health and Education Research Ethics and Governance Committee at Manchester Metropolitan University granted ethical approval (Project ID, 47784).

## Results

### Analysis

The researchers conducted analyses of direct relationships and differences using SPSS v28 and performed latent profile analysis (LPA) with Mplus v8 (Muthén and Muthén, 2018). LPA statistically groups participants using their response patterns to selected variables. In the present study, the variables were the level of paranormal belief and schizotypy. Predetermined criteria identified the optimal number of latent profiles, specifically the Akaike information criterion (AIC; Akaike, 1987), sample size-adjusted BIC (ssaBIC; Sclove, 1987), the Lo–Mendell–Rubin-adjusted likelihood ratio test (LMR-A-LRT; Lo et al., 2001), and a test of entropy (Ramaswamy et al., 1993).

Lower AIC and ssaBIC denote superior fit, and entropy values >0.8 represent the sound division of profiles. The LPA process iteratively tests models with an increasing number of profiles (commencing at one), until non-significant improvement occurs. The LMR-A-LRT and its *p*-value determined whether a profile model fitted data significantly better than a competing solution. Multivariate analyses of variance (MANOVA) then assessed whether latent profiles differed significantly on outcome variables: paranormal experience, PE; belief in conspiracies, GCB-5; meaning in life, presence (MLPresence) and search (MLSearch), and satisfaction with life, present (SWLS); and self-esteem.

### Primary analysis

Normality assessment revealed that RPBS and SPQ-B skewness and kurtosis values were within the acceptable range of −2 to +2 (Byrne, 2013). Pearson correlation revealed small to large (see Gignac and Szodorai, 2016) positive, significant relationships between RPBS and SPQ-B subscales of cognitive–perceptual (*r* = 0.54, ‘relatively large’), interpersonal (*r* = 0.16, ‘relatively small’), and disorganised (*r* = 0.25, typical). Moreover, RPBS correlated positively with PE (dummy coded), GCB-5, MLPresence, MLSearch, and SWLPresent. All SPQ-B subscales correlated positively with PE, GCB-5, and MLSearch and negatively with MLPresence, SWLPresent, and Self-Esteem (Table 1).

**Table 1:** Correlations amongst paranormal belief and schizotypy.

Variable	Mean	*SD*	1	2	3	4	5	6	7	8	9	10	11
1. RPBS	89.13	34.44		0.38^**^	0.54^**^	0.16^**^	0.25^**^	0.32^**^	0.57^**^	0.14^**^	0.36^**^	0.05^*^	−0.01
2. SPQ-B	8.61	5.47			0.82^**^	0.83^**^	0.82^**^	0.24^**^	0.36^**^	−0.20^**^	0.34^**^	−0.24^**^	−0.30^**^
3. Cognitive–perceptual	2.75	2.30				0.46^**^	0.56^**^	0.33^**^	0.45^**^	−0.04	0.37^**^	−0.13^**^	−0.14^**^
4. Interpersonal	4.16	2.50					0.56^**^	0.08^**^	0.20^**^	−0.26^**^	0.20^**^	−0.27^**^	−0.34^**^
5. Disorganised	1.70	1.82						0.20^**^	0.25^**^	−0.19^**^	0.27^**^	−0.17^**^	−0.25^**^
6. PE	0.08	0.26							0.20^**^	0.10^**^	0.15^**^	0.08^**^	0.07^**^
7. GCB-5	15.22	4.77								0.02	0.27^**^	−0.03	−0.03
8. MLPresence	21.60	6.71									−0.01	0.58^**^	0.62^**^
9. MLSearch	21.46	7.01										−0.14^**^	−0.13^**^
10. SWLS	20.34	7.79											0.57^**^
11. Self-esteem	17.45	3.73											

Raw means: RPBS, Revised Paranormal Belief Scale; SPQ-B, Schizotypy Personality Questionnaire—Brief; PE, Paranormal Experience; GCB-5, Generic Conspiracist Beliefs Scale—Short; MLPresence, meaning in life presence; MLSearch, search for meaning; SWLS, Satisfaction with Life Scale; Self-Esteem, Rosenberg Self-Esteem Scale; ^*^p < 0.05, ^**^p < 0.001.

### Latent profile analysis

**Figure 1:**
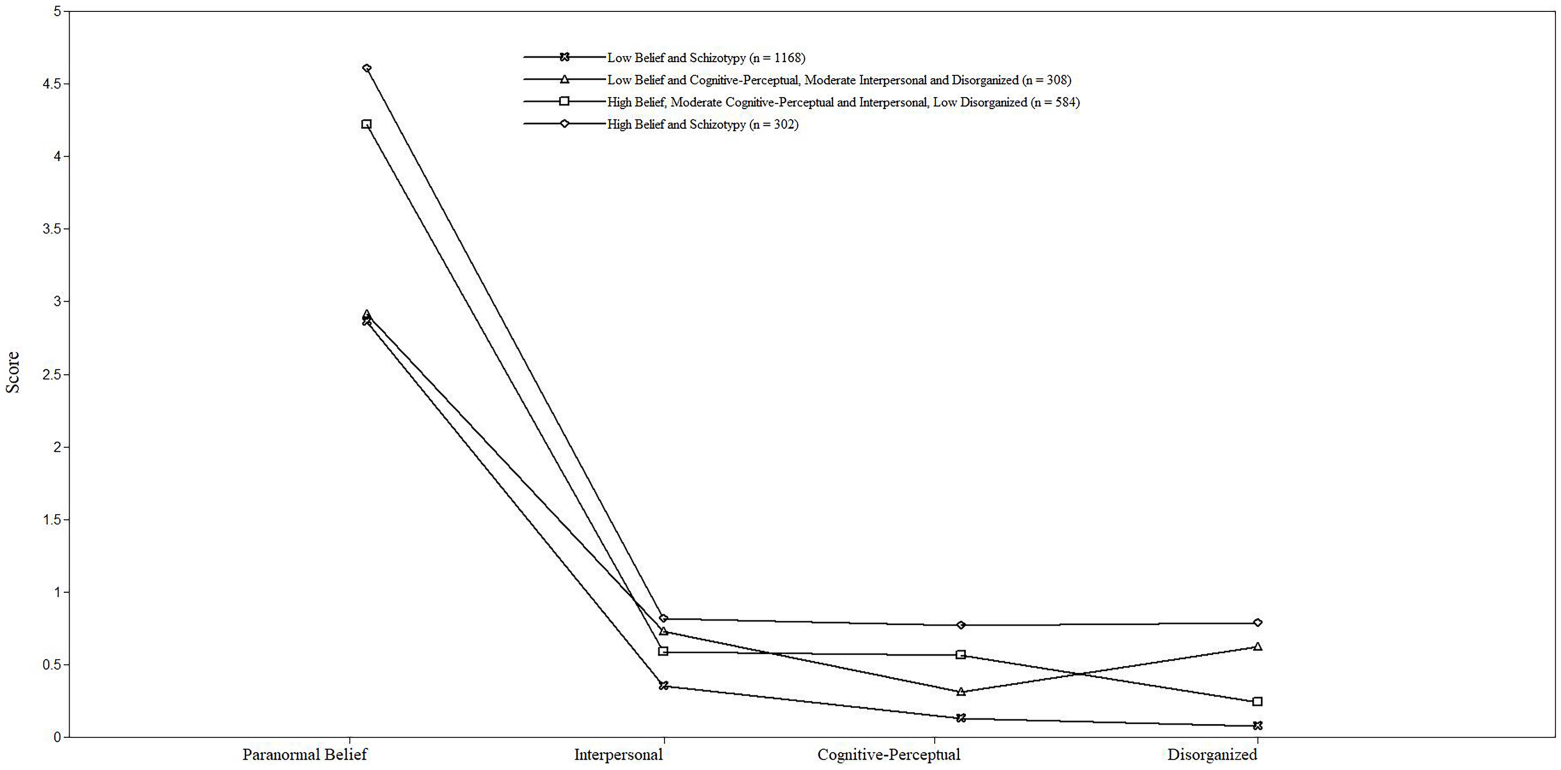
Pattern of mean scores on the Revised Paranormal Belief Scale (RPBS) and Schizotypy Personality Questionnaire—Brief (SPQ-B) as a function of latent profile.

Using RPBS and SPQ-B subscale mean scores, fit indices specified that a four-profile solution was superior to competing models, lower AIC and ssaBIC (vs. three-profile solution), and greater entropy (vs. five-profile solution) existed. Additionally, the five-profile solution produced a non-significant LMR-A-LRT *p-*value (Table 2). Figure 1 shows a four-profile solution subgroup constituency. Profiles reflected relative differences in scoring: Profile 1 (low belief and schizotypy, 49% of sample); Profile 2 (low belief and cognitive–perceptual, moderate interpersonal and disorganised, 13.6%); Profile 3 (high belief, moderate cognitive–perceptual and interpersonal, low disorganised, 24.3%); and Profile 4 (high belief and schizotypy, 13.1%). Within the mixed schizotypy subgroups, Profile 2 displayed moderate levels of interpersonal and disorganised and Profile 3 displayed moderate levels of cognitive–perceptual and interpersonal.

**Table 2:** Fit of latent profile solutions.

Model	AIC	ssaBIC	LMR-A	LMR-A *p-*value	Entropy
1-class	11164.77	11185.49			
2-class	8934.46	8968.14	2184.06	< 0.001	0.83
3-class	8279.88	8326.50	647.90	< 0.001	0.86
4-class	7793.17	7852.74	484.24	< 0.001	0.83
5-class	7579.07	7651.59	218.47	0.085	0.81

AIC, Akaike information criterion; ssaBIC, sample size-adjusted BIC; LMR-A, Lo–Mendell–Rubin-adjusted likelihood ratio test.

Conditional response means (Appendix Table S1) indicated that within paranormal belief, scores varied across profiles (i.e., Profiles 1 and 2 displayed < mean; Profiles 3 and 4 displayed > mean). Within schizotypy, except for Profiles 1 and 2 (consistently low and high scores), less profile variation occurred. Profile 2 exhibited < mean on cognitive–perceptual, and Profile 2 exhibited > mean on interpersonal and disorganised. Profile 3 exhibited > mean on cognitive–perceptual and interpersonal, and Profile 3 exhibited < mean on disorganised.

### Profile variations in paranormal experience, conspiracy belief, and well-being

MANOVA (Table 3) found a significant medium-sized effect, Pillai’s trace = 0.36, *F*(18, 7,065) = 53.33, *p* < 0.001, η^2^ = 0.12. On individual measures, there were significant effects on PE (with high scores as the reference category), *F*(3, 2,358) = 77.78, *p* < 0.001, η^2^ = 0.09 (medium effect); GCB-5, *F*(3, 2,358) = 180.53, *p* < 0.001, η^2^ = 0.19 (large effect); MLPresence, *F*(3, 2,358) = 38.35, *p* < 0.001, η^2^ = 0.05 (small effect); MLSearch, *F*(3, 2,358) = 117.48, *p* < 0.001, η^2^ = 0.13 (medium effect); SWLS, *F*(3, 2,358) = 28.32, *p* < 0.001, η^2^ = 0.04 (small effect); and Self-Esteem, *F*(3, 2,358) = 48.56, *p* < 0.001, η^2^ = 0.06 (medium effect).

**Table 3:** Relationships of the group (latent profile) with study outcomes.

	Dependent variable								
	PE	GCB-5	MLPresence	MLSearch	SWLS	Self-esteem			
	ANOVA							MANOVA	
	*F^df^* (*Sig.*; η^2^)	*F^df^* (*Sig.*; η^2^)	*F^df^* (*Sig.*; η^2^)	*F^df^* (*Sig.*; η^2^)	*F^df^* (*Sig.*; η^2^)	*F^df^* (*Sig.*; η^2^)	Pillai	*F^df^* (*Sig.*)	η^2^
**Variable**									
Group	77.78 ^3,2,358^ (<0.001; 0.09)	180.53 ^3,2,358^ (<0.001; 0.19)	38.35 ^3,2,358^ (<0.001; 0.05)	117.48 ^3,2,358^ (<0.001; 0.13)	28.32 ^3,2,358^ (<0.001; 0.04)	48.56 ^3,2,358^ (<0.001; 0.06)	0.36	53.33 ^18,7,065^ (<0.001)	0.12
	**Pairwise comparisons (mean differences) between profiles**								
**Profile contrast**	**Mean diff. (*Sig.*)**	**Mean diff. (*Sig.*)**	**Mean diff. (*Sig.*)**	**Mean diff. (*Sig.*)**	**Mean diff. (*Sig.*)**	**Mean diff. (*Sig.*)**			
Profile 1 vs. Profile 2	−0.03 (0.306)	−1.28 (<0.001)	4.39 (<0.001)	−2.34 (<0.001)	3.94 (<0.001)	2.31 (<0.001)			
Profile 1 vs. Profile 3	−0.11 (<0.001)	−3.86 (<0.001)	0.55 (0.562)	−4.48 (<0.001)	2.07 (<0.001)	0.86 (<0.001)			
Profile 1 vs. Profile 4	−0.22 (<0.001)	−5.31 (<0.001)	1.65 (<0.001)	−6.80 (<0.001)	2.62 (<0.001)	2.03 (<0.001)			
Profile 2 vs. Profile 3	−0.08 (<0.001)	−2.57 (<0.001)	−3.83 (<0.001)	−2.14 (<0.001)	−1.87 (0.003)	−1.44 (<0.001)			
Profile 2 vs. Profile 4	−0.19 (<0.001)	−4.02 (<0.001)	−2.73 (<0.001)	−4.46 (<0.001)	−1.32 (0.199)	−0.27 (1.00)			
Profile 3 vs. Profile 4	−0.10 (<0.001)	−1.45 (<0.001)	1.10 (0.107)	−2.31 (<0.001)	0.54 (1.00)	1.17 (<0.001)			

Profile 1, low belief and schizotypy; Profile 2, low belief and cognitive–perceptual, moderate interpersonal and disorganised; Profile 3, high belief, moderate cognitive–perceptual and interpersonal, low disorganised; Profile 4, high belief and schizotypy; PE, Paranormal Experience; GCB-5, Generic Conspiracist Beliefs Scale—Short; MLPresence, meaning in life presence; MLSearch, search for meaning; SWLS, Satisfaction with Life Scale; Self-Esteem, Rosenberg Self-Esteem Scale.

*Post-hoc* pairwise comparisons (rescaled to significance at *p* < 0.05 using Bonferroni) assessed between-profile differences. Regarding MLPresence, Profile 2 scored lower than other subgroups (Profiles 1, 3, and 4). Additionally, Profile 1 scored higher than Profile 4. This indicated that low belief with mixed schizotypy was associated with lower presence, whereas low belief and schizotypy (vs. high) were related to higher MLPresence. For MLSearch, higher levels were evident in Profile 4 (vs. Profiles 1, 2, and 3), Profile 3 (vs. Profiles 2 and 1), and Profile 2 vs. Profile 1. These differences indicated that greater MLSearch was associated with a higher presence of paranormal belief and schizotypy.

Regarding SWLS, Profile 1 (vs. Profiles 2, 3, and 4) and Profile 3 (vs. Profile 2) demonstrated greater SWLS. The pattern of responses for self-esteem was the same with the addition that Profile 4 (vs. Profile 3) scored higher. Lower belief and schizotypy were associated with higher satisfaction with life and self-esteem. Additionally, amongst participants with moderate schizotypy (Profile 3 vs. Profile 2), higher belief was related to higher satisfaction with life and self-esteem.

For PE and GCB-5, Profile 4 (vs. Profiles 3, 2, and 1), Profile 3 (vs. Profiles 2 and 1), and Profile 2 vs. Profile 1 scored higher. The levels of paranormal belief and schizotypy were associated with reporting of experiences and endorsement of conspiracy beliefs; higher belief and schizotypy showed greater reporting of experiences and endorsement of generic conspiracy beliefs.

## Discussion

Consistent with prior research, paranormal belief correlated positively with schizotypy and was most strongly related to the cognitive–perceptual factor (Darwin et al., 2011). This relationship was attributable to overlapping construct features (i.e., odd/magical beliefs, unusual perpetual experiences, and ideas of reference). Paranormal belief and the cognitive–perceptual factor were associated with higher scores on reporting paranormal experiences and endorsement of generic conspiracist beliefs, respectively. Despite commonality, paranormal belief and schizotypy were differentially related to well-being measures. Paranormal belief correlated positively with meaning in life (presence and search) and satisfaction with life, whereas schizotypy correlated negatively with presence, satisfaction with life, and self-esteem and positively with search.

The observation that paranormal belief was associated with higher presence, whilst schizotypy was associated with lower scores was important in the context of well-being. Presence correlates positively with affirming outcomes (e.g., life satisfaction and joy) and negatively with adverse indicators (e.g., depression and sadness; Steger et al., 2006). Although the relationship between presence and search is complex and academically debated (Newman et al., 2018), search typically associated with negative emotions (Cohen and Cairns, 2012) and reduced well-being (e.g., depression, sadness, and rumination; Dakin et al., 2021). As paranormal belief and schizotypy were both positively related to search, their distinct relationship with presence was potentially important. In the case of believers, high presence could mediate relationships between search and negative well-being outcomes (Newman et al., 2018). Future studies should assess these relationships, employing a broader set of well-being outcomes.

Regarding satisfaction with life and self-esteem, schizotypy was associated with lower scores, and paranormal belief was weakly positively related with satisfaction with life. There was no significant correlation between paranormal belief and self-esteem. Overall paranormal belief was associated with positive well-being, and schizotypy was associated with lower scores on positive well-being measures. This was ascribed to the interpersonal and disorganised factors, which reflect affective and social deficiencies (psychological maladjustment) that are inclined to undermine positive well-being (Mohr and Claridge, 2015).

The present study also observed that the cognitive–perceptual schizotypy factor correlated negatively with satisfaction with life. This finding was commensurate with the supposition that the effects of positive schizotypy are contingent on concurrent levels of interpersonal (negative) and disorganised factors (Abbott and Byrne, 2012). This is because positive schizotypy is more strongly related to negative affect than life satisfaction (Abbott et al., 2012). Indeed, positive schizotypy is a weak predictor of life satisfaction (Abbott and Byrne, 2012). In the context of the present study, it appears that levels of interpersonal (negative) and disorganised factors (especially the former) and allied negative affect/absence of positive affect influenced cognitive appraisal of well-being (i.e., satisfaction with life and self-esteem). This explains why overall schizotypy is associated with diminished life satisfaction (Abbott et al., 2012).

Associations and their implications require cautious interpretation. For example, whilst the relationship between paranormal belief and life satisfaction was significant, the effect size was small. This concurred with prior studies reporting weak to non-significant associations (e.g., Dagnall et al., 2022a,b). Indeed, paranormal belief demonstrates (negative) relationships via the influence of attendant constructs, such as transliminality. The significant paranormal belief–meaning in life relationship has more robust support (cf. FioRito et al., 2021; Dagnall et al., 2022a), indicating a direct (and consistent) link with well-being.

Profile comparisons concurred with the analysis of direct relationships. Low belief and cognitive–perceptual, moderate interpersonal and disorganised (Profile 2) was associated with lower MLPresence (vs. Profiles 1, 2, and 4), and low belief and schizotypy (Profile 1) (vs. high, Profile 4) was related to higher MLPresence. Generally, paranormal belief and schizotypy (Profiles 2, 3, and 4) were associated with greater MLSearch, higher scores on paranormal experiential factors, and endorsement of generic conspiracist beliefs. Finally, low belief and schizotypy (Profile 1) were concomitant with higher satisfaction with life and self-esteem.

Conclusions derived from the profiles require cautious interpretation. Although LPA produces statistically sound subgroups, these were not theoretically informed. Instead, labels reflected characteristics from the observed sample. This was not problematic in the current study as it was exploratory and focused on differences arising from the heterogeneous nature of belief, particularly examining whether interactions with schizotypy affected scores on positive well-being outcomes. To enable comparisons across studies, investigators need to conduct subsequent scholarly work that identifies robust profiles. This is problematic with LPA because subgroups arise from cross-variable heterogeneity within a particular model. Hence, profiles may vary across samples. In this context, replications and cross-validation using techniques, such as progressive elaboration, are necessary (Donovan and Chung, 2015). This iterative approach establishes model fit and class constancy, enabling researchers to produce conceptually valid profiles.

There are also other study limitations that merit consideration. The use of cross-sectional designs (i.e., collecting data at one point in time) is often criticised because single measurements provide only estimates of complex effects, which may change over time. To examine variations and establish measurement stability, longitudinal studies with multiple points are necessary. This would indicate whether paranormal belief has long-term benefits and determine whether cognitive–perceptual and personality factors influence effects. In this context, it is also important to reproduce the results of the study with independent samples. Additionally, to ensure that outcomes were not context-specific, this should extend to assessing generalisability across societies. This will help to determine whether cultural variations influence belief adaptivity.

Furthermore, self-report measures are potentially problematic because they assume that respondents can accurately assess their beliefs and well-being and/or respond honestly. Additionally, this study used a narrow set of well-being outcomes. Acknowledging this, future studies should employ objective measures and a broader range of factors. This will enable researchers to look more generally at the potential benefits of belief. Recognising this, subsequent investigations could include the Quality of Life Scale (Burckhardt and Anderson, 2003), which measures five conceptual domains: material and physical well-being; relationships with other people; social, community, and civic activities; personal development and fulfilment; and recreation. This will provide a broad multidimensional assessment of perceived personal well-being.

Due to the exploratory nature of this study and the need to restrict variable numbers for analysis, profiles are derived from unidimensional measures of paranormal belief. Future investigations should examine whether facets of credence are differentially related to well-being. For instance, the bifactor RPBS structure suggests that New Age Philosophy (NAP) and Traditional Paranormal Belief (TPB) reflect different mechanisms for exerting control over external events. NAP provides a sense of control at the individual level (Irwin, 2004), whilst TPB achieves this at a social level.

Given the complex nature of the reported effects, the practical significance to clinical and real-world settings is hard to determine (Achterhof et al., 2019). From a conceptual standpoint, the study represents an important starting point for understanding relationships between paranormal belief, schizotypy, and positive well-being.

## Data availability statement

The raw data supporting the conclusions of this article will be made available by the authors, without undue reservation.

## Ethics statement

The studies involving humans were approved by the Health and Education Research Ethics and Governance Committee at Manchester Metropolitan University granted ethical approval (Project ID, 47784). The studies were conducted in accordance with the local legislation and institutional requirements. The participants provided their written informed consent to participate in this study.

## Author contributions

ND: Conceptualization, Data curation, Formal analysis, Funding acquisition, Investigation, Methodology, Resources, Writing – original draft, Writing – review & editing. KD: Conceptualization, Funding acquisition, Investigation, Writing – original draft, Writing – review & editing. AD: Conceptualization, Formal analysis, Investigation, Writing – original draft, Writing – review & editing. AE: Conceptualization, Writing – review & editing.
