# Supplementary material for: Variations in positive well-being as a function of the interaction between paranormal belief and schizotypy
Source: Front Psychol. 2024 Jul 26;15:1396485. doi: 10.3389/fpsyg.2024.1396485 (PMC11310046; doi:10.3389/fpsyg.2024.1396485)
Supplement: Supplementary file 1 [file Table_1.DOCX]

**Appendix S1** Conditional response means among latent profiles

| Scale | Scale mean | Profile 1: Low Belief and Schizotypy | Profile 2: Low Belief and Cognitive-Perceptual, Moderate Interpersonal and Disorganized | Profile 3: High Belief, Moderate Cognitive-Perceptual and Interpersonal, Low Disorganized | Profile 4: High Belief and Schizotypy |
| --- | --- | --- | --- | --- | --- |
| Paranormal Belief | 3.42 | 2.86 | 2.91 | 4.22 | 4.61 |
| Cognitive-Perceptual | 0.34 | 0.13 | 0.31 | 0.57 | 0.77 |
| Interpersonal | 0.52 | 0.35 | 0.73 | 0.59 | 0.81 |
| Disorganized | 0.28 | 0.07 | 0.63 | 0.24 | 0.79 |
